# Comparing the Prevalence of Polypharmacy and Potential Drug-Drug Interactions in Nursing Homes and in the Community Dwelling Elderly of Emilia Romagna Region

**DOI:** 10.3389/fphar.2020.624888

**Published:** 2021-02-11

**Authors:** Sofia Burato, Luca Leonardi, Ippazio Cosimo Antonazzo, Emanuel Raschi, Chiara Ajolfi, Manuela Baraghini, Antonella Chiarello, Valentina Delmonte, Lucio Di Castri, Monia Donati, Antonella Fadda, Daniela Fedele, Alessandra Ferretti, Laura Gabrielli, Silvia Gobbi, Sereno Lughi, Martina Mazzari, Fabio Pieraccini, Alessandro Renzetti, Elsa Russi, Chiara Scanelli, Barbara Zanetti, Elisabetta Poluzzi

**Affiliations:** ^1^ Department of Medical and Surgical Sciences, University of Bologna, Bologna, Italy; ^2^ Post Graduate School of Hospital Pharmacy, Department of Pharmacy, University of Pisa, Pisa, Italy; ^3^ Local Health Authority of Modena, Modena, Italy; ^4^ Local Health Authority of Romagna (Forlì and Cesena Units), Emilia-Romagna, Italy; ^5^ Local Health Authority of Imola, Imola, Italy; ^6^ Local Health Authority of Parma, Parma, Italy; ^7^ Local Health Authority of Piacenza, Piacenza, Italy; ^8^ Local Health Authority of Bologna, Bologna, Italy; ^9^ Local Health Authority of Ferrara, Ferrara, Italy; ^10^ Local Health Authority of Reggio Emilia, Reggio Emilia, Italy; ^11^ University Hospital of Ferrara, Ferrara, Italy; ^12^ Centre of Studies and Research on the Elderly, University of Bologna, Bologna, Italy

**Keywords:** drug-drug interactions (DDIs), older peolpe, drug utilization, inappropriateness, nursing home, community dwelling

## Abstract

**Backround:** We aimed at assessing the prevalence of polypharmacy and potential drug-drug interactions (DDIs) with clinical relevance in elderly patient on Emilia Romagna area. Both outpatients and residents in nursing homes were assessed, with only partially overlapping strategies.

**Methods:** We defined a list of 190 pairs of potentially interacting drugs, based on literature appraisal and availability of therapeutic alternatives. January-June 2018 data on drug use in patients over 65 years-old were collected from nine Local Health Authorities of Emilia Romagna: data on community-dwelling subjects were extracted from archives of reimbursed prescriptions, while drug use in a sample of nursing homes was recorded from clinical charts in one index day within the same semester. The frequency of polypharmacy (at least five or at least 10 concurrent drugs) and of each DDI was calculated.

**Results:** In line with different rates of polypharmacy (80% vs 16%), the risk of exposure to at least one interaction was 53.7% in nursing homes and 26.4% in outpatients. Among DDIs, in nursing homes antidepressants—anxiolytics (11.9%) ranked first, followed by antidepressants—aspirin (7.4%). In outpatients, ACE-inhibitors—non-steroidal anti-inflammatory drugs (NSAIDs) reached 7.2% followed by the calcium channel blockers—α-blockers (2.4%).

**Discussion:** Polypharmacy and risk of DDIs appeared very different in the two settings, due to both technical and clinical reasons. In order to reduce use of benzodiazepines, NSAIDs, antidepressants and relevant DDIs, 1) defining alternative options for pain relief in elderly outpatients, and 2) implementing non-pharmacological management of insomnia and anxiety in nursing homes should be prioritized.

## Introduction

According to the most recent reports released by the WHO (World Health Organization, 2015), the global prevalence of people over 65 years of age is growing rapidly, and it is assumed that by 2050 it will increase from 12 to 22%. Elderly patients represent a challenge for clinicians because of both age-related physiological changes, which make patients extremely susceptible to the effects of drug therapy, and the presence of several comorbidities, with relevant difficulties in managing drug polypharmacy.

In terms of clinical outcomes, the main concerns are represented by an increasing risk of adverse drug reactions (ADRs) and lack of effectiveness, especially due to Drug-Drug Interactions (DDIs) and impaired compliance.

Polypharmacy is defined as the concurrent use of multiple medicines. Risk of DDIs and relevant ADRs significantly increases with increasing polypharmacy. Although it is not simply a synonym of inappropriate drug use (Cadogan et al., 2016; Hughes, 2020), a defined threshold is still considered useful to identify patients to be primarily addressed to prescription reviewing process (Viktil et al., 2007; Onder et al., 2010; Masnoon et al., 2017).

From an epidemiological point of view, the prevalence of potential DDIs in the Elderly has been roughly estimated between 35 and 60%, of which 5–15% are responsible for adverse reactions, mostly avoidable or manageable (Magro et al., 2012; Obreli-Neto et al., 2012), with a remarkable prevalence of hospital admission due to interactions (>1%) (Dechanont et al., 2014; Bénard-Laribière et al., 2015).

Although identification of the actual role of DDIs in severe events (e.g., leading to hospitalization) in this specific population is challenging, the risk of specific DDIs has been already confirmed by clinical data (Vazquez, 2018; Jobski et al., 2020; Swart et al., 2020).

Innovative strategies to reduce the frequency of potential DDIs and the incidence of related ADRs are increasingly tested in clinical practice. Pharmacist-led interventions (e.g. medication reviews, consultations with patient and physician) demonstrated an added value in managing at risk polypharmacy, although costs are not easily affordable by public or private payers (Desmaele et al., 2015; Rankin et al., 2018; Quintens et al., 2019). Different mobile health apps offer a lot of options for prescriber and even patient self-assessment (DDI checker, medication reminder, refill reminder, medication history tracking and pill identification), although lack of complete information or obstacles in their use can strongly affect their performance (Kim et al., 2018).

Elderly patients are encountered in different settings of care: in primary care (community-dwelling) or in nursing homes for the management of chronic conditions, and at hospital only for acute conditions. Nursing home residents typically include older people with a professional support in monitoring healthcare status and helping in adhering to drug therapy. This setting represents a partially neglected/under-explored area of research where real-world studies should be encouraged (Pasina et al., 2020). Community dwelling subjects are usually healthier and younger, but they cannot rely on the intensive help of health professionals. As a matter of fact, each setting requires specific analysis of possible ad hoc strategies to be implemented toward supporting patients in avoiding the adverse consequences of DDIs.

The present work has set itself the objective of evaluating what the dimension of clinically relevant drug-drug interactions is in elderly patients out of hospital, by assessing two different sub-populations: community dwelling subjects and residents in nursing homes. The final aim is to identify specific drug-drug interactions on which, in a specific setting, improvement strategies should be implemented.

## Methods

### Source of Data and Study Population

We conducted an observational study on the geriatric population of the Emilia-Romagna Region as a part of a pharmacovigilance project funded by the Italian Medicines Agency (Agenzia Italiana del Farmaco—AIFA) and the Emilia Romagna Regional Health Authority for post-marketing activities, aiming at increasing knowledge of ADRs and improving appropriateness of drug prescription.

For this purpose, we analyzed drug prescription during 2018 in two different settings: 1) a cohort of about 835,000 community-dwelling older people (≥65) assisted by 9 out of 11 Local Health Authorities (LHAs) of Emilia Romagna Region (Piacenza, Parma, Reggio Emilia, Modena, Ferrara, Bologna, Imola, Forlì and Cesena), with a reference population of about 4.5 million inhabitants; and 2) about 4,000 elderly people institutionalized in a sample of nursing homes of the same LHAs. For each patient, the presence of polypharmacy and specific pairs of drugs with potential interactions (drug-drug interactions—DDIs) was assessed.

According to the most accepted definitions, we considered polypharmacy as concurrent use of either at least five different medications (broad definition) or at least 10 different medications (narrow definition) (Gnjidic et al., 2012).

As for DDIs, by starting from the analysis of several lists published in the literature and mainly referring to a previous approach used by our group (Raschi et al., 2015), a multidisciplinary panel of experts (comprising pharmacists, clinical pharmacologists, and pharmacovigilance experts of the nine participant LHAs) compiled a list of clinically important drug-drug pairs. The list was developed to be as comprehensive as possible, but only manageable interactions were included (i.e., avoidable by switching to alternative therapeutic options or strictly monitorable by *ad hoc* tests) and at least one drug usually taken for chronic purposes. The components in the drug-drug interaction may be a drug class or a single active substance depending on the available evidence. Supplementary Tables S1, S2 show the 190 DDIs, with relevant mechanisms of interaction, clinical effects, therapeutic alternatives, and/or monitoring strategies for early prediction of effects.

For the community dwelling cohort, we selected all subjects 65 and older with at least one reimbursed prescription in the first semester 2018. The LHA administrative healthcare database contains information on insured subjects (unique identification number, sex, and age), on reimbursed prescriptions (drug names, trade names, claim date, number of packages dispensed, and Anatomical Therapeutic Chemical Classification System [ATC] code) (WHO Collaborating Center for Drug Statistics Methodology, 2019) and on prescribers (identifier numbers and specialty). Subjects were considered under polypharmacy if they chronically received at least five drugs (broad) or at least 10 drugs (narrow) in the analyzed semester. Chronic use was defined as coverage of more than half a semester, i.e., >90 DDDs (Defined Daily Doses) (WHO Collaborating Center for Drug Statistics Methodology, 2019), except for some classes for which a lower threshold was considered (e.g., antibiotics, NSAIDs, and corticosteroids; see supplementary material). Similarly, patients were considered under interaction when both drugs were prescribed in the analyzed semester, provided that one drug of the pair covered more than 90 days (DDDs >90).

As regards nursing homes, a sample of residents (about 400) was selected for each participant LHA. For a specific index date, personal data of each patient, administered drug treatments and relevant indications of use were extracted from clinical charts and recorded on a dedicated server. For both polypharmacy and interactions, relevant drugs should be taken in the same day.

### Data Analysis

The study envisaged the use of a dedicated server for data storage and processing. The data mining procedures were performed using the free software R-Studio, PostgreSQL, and Access of the Microsoft Office package.

Descriptive statistics were carried out to describe the baseline characteristics of the study population.

The frequency of polypharmacy, as well as each potential interaction, was obtained for each setting of care from the percentage ratio between patients exposed to polypharmacy, or each specific interaction respectively, and the total number of patients in the cohort.

Differences of population characteristics between nursing home and community-dwelling cohorts were tested with chi-square tests for categorical variables and Student’s t-tests for continuous variables.

### Ethical Issues

This retrospective study was carried out according to the regulations on data management with the Italian law on privacy (Legislation Decree 196/2003 amended by Legislation Decree 101/2018). Pseudonymized administrative data can be used without a specific written informed consent when patient information is collected for healthcare management and healthcare quality evaluation and improvement (according to art. 110 on medical, biomedical, and epidemiological research, Legislation Decree 101/2018).

In the present study, data were pseudonymized by each single LHA, analyzed, and aggregated at the local level. Aggregated data were then shared among participants to the project in order to obtain overall frequencies of each indicator described above. The Italian Medicines Agency (Agenzia Italiana del Farmaco—AIFA) and the Emilia Romagna Regional Health Authority, which funded this study as part of a healthcare quality improvement initiative, were informed on this publication.

## Results

The nursing home cohort included 3,946 institutionalized patients: 70.4% were women and the mean age was 84.4 years (subjects over-80s were 72.0%). The outpatient cohort included 835,247 subjects (i.e. 94.7% of the inhabitants ≥65 insured by the public drug plan of the nine LHAs): 56.9% were women and the mean age was 77.0 years; the over 80 s were 31.4%.

Polypharmacy with ≥5 drugs was very common in all nursing homes with an average regional prevalence of 80.3%, whereas among elderly outpatients it was only 16.1%. Again, ¼ of subjects residing in nursing homes (23.8%) received at least 10 concomitant drugs, while among community dwelling this rate was 0.5% (Table 1).

**TABLE 1:** Demographic characteristics of selected cohort.

Characteristics	Inpatients in nursing homes (*N* = 3,946)	Outpatients (*N* = 835,247)	*p* value
Sex			<0.001
Male, *N* (%)	1,169 (29.6)	359,686 (43.1)	
Female, *N* (%)	2,777 (70.4)	475,566 (56.9)	
Age. Mean (SD)	84.4 (1.4)	77.0 (0.9)	
Age class			<0.001
<65, *N* (%)	51 (1.4)	—	
65–70, *N* (%)	187 (4.7)	220,781 (26.4)	
71–75, *N* (%)	299 (7.5)	178,477 (21.4)	
76–80, *N* (%)	551 (14.1)	173,454 (20.8)	
> 80, *N* (%)	2,862 (72.3)	262,628 (31.4)	
Polypharmacy			<0.05
5–9 drugs, *N* (%)	2,236 (56.5)	130,771 (15.6)	
≥10 drugs, *N* (%)	956 (23.8)	3,765 (0.5)	
Risk of potential interactions to at least one couples, *N* (%)	2,119 (53.7)	220,175 (26.4)	

In nursing homes, a total of 190 pairs of interacting drugs were analyzed, resulting in a frequency of 53.7% (Table 2 shows the most common interactions, while the entire list is part of the supplementary material). Among community-dwelling subjects the 150 interactions including only reimbursed medicines amounted to 26.4%. If from the list of interactions analyzed in nursing homes, we exclude the 40 pairs of interactions (mainly formed by benzodiazepines) that cannot be studied among the dwelling communities (not reimbursed medicines), this risk of exposure decreases to 32.4%.

**TABLE 2:** Prevalence of potential drug-drug Interactions by therapeutic drug class and individual agents. Only pairs with a prevalence ≥ 1% are listed.

Inpatients in nursing homes (*N* = 3,946)		Outpatients (*N* = 835,247)	
Drug-drug interaction	Prevalence	Drug-drug interaction	Prevalence
1. Antidepressants—Anxiolytics	11.9%	1. ACEIs/sartans—NSAIDs	7.2%
2. SSRIs—ASAs	7.4%	2. Calcium channel blockers—α-blockers/α-adrenoreceptor agonists	2.4%
3. Antidiabetics—β-adrenoceptors blockers	5.3%	3. Antidiabetics—β-adrenoceptors blockers	2.3%
4. Vitamin K antagonists—PPIs	4.1%	4. SSRIs—ASAs	2.2%
5. Anxiolytics, benzodiazepines—opioids	3.4%	5. Diuretics—NSAIDs	1.3%
6. Anxiolytics, benzodiazepines—hypnotics	2.8%	6. SSRIs—NSAIDs	1.1%
7. Allopurinol—ACEIs	2.7%	7. Antidiabetics—Fluoroquinolones	1.0%
8. Antidepressants—Fentanyl	2.5%	8. Calcium channel blockers—macrolides	1.0%
9. Vitamin K antagonists—SSRIs	2.1%	—	
10. Calcium channel blockers—α-blockers/α-adrenoreceptor agonists	1.6%	—	
11. Anxiolytics, benzodiazepines—Antihistamines	1.4%	—	

The most frequent co-prescription of potentially interacting drugs in nursing homes was represented by antidepressants—anxiolytics (11.8%), followed by SSRIs—ASA (7.3%) and antidiabetics—beta-blockers (5.3%).

As far as the community-dwelling prescriptions are concerned, the most frequent pair was ACE-inhibitors—NSAIDs (7.2%), followed by calcium channel blockers—α-blockers (2.4%; including both antihypertensive and drugs used in benign prostatic hypertrophy) and by antidiabetics—beta-blockers (2.3%).

Classes of drugs most involved in potential interactions were antidepressants, anxiolytics and vitamin K antagonists for nursing home setting, and NSAIDs, antidiabetics, SSRIs, and calcium channel blockers for the community-dwelling population.

No correlation of prevalence of DDIs and polypharmacy between nursing homes and community dwelling setting within the same LHA was found.

## Discussion

To the best of our knowledge, this is the first Italian population-based study providing an overall picture of polypharmacy and DDIs in a sample of the Italian geriatric population, by monitoring both community-dwelling and nursing-home institutionalized subjects. The two mentioned populations differ in health status and level of care: the former live at home, refer mainly to general practitioner and in some cases are supported by relatives or caregivers, while residents in nursing homes have more complex clinical profiles and a more intensive level of care. This last aspect allows closer monitoring of drug effect and treatment adherence, but probably induces the use of a greater number of drug treatments, as documented by our findings: i.e., polypharmacy is much more common in nursing homes than in the community-dwelling population (80 vs. 16%). The number of concomitant drugs is considered the most important predictive factor of ADR by the literature with a linear relationship with the risk of adverse events (Viktil et al., 2007); general reduction of medications in nursing homes is therefore strongly recommended for a safer drug use.

Over 1/3 of both populations (by excluding psychotropic medicines, which are mapped only in nursing homes) was exposed to at least one drug-drug interaction; this finding is similar to the recent study by Hanlon et al (Hanlon et al., 2017) on well-functioning community-resident adults aged 70–79 years. In our study, out of 190 potential drug interactions detectable in nursing homes, 10 resulted in a frequency of use of >1%, while out of 150 pairs studied among community-dwelling patients, 9 emerged as common.

Pharmacodynamic mechanisms have been identified as the main underlying pharmacological basis and bleeding and dysglycemia are potentially the most frequent ADRs.

The first ranked interaction was antidepressants with anxiolytics in nursing homes (>1 out of 10 residents); its risk is a strong sedative effect (Kanba, 2004) which, especially in the Elderly, can allow for an increased rate of falls and relevant fractures (Vestergaard et al., 2006; Aizenberg et al., 2015). Many additional interactions between psychotropic medications with high risk of sedation and consequent falls were found in >1% of nursing home residents: benzodiazepines—opioids, benzodiazepines—hypnotics, antidepressants—fentanyl, benzodiazepines—antihistamines.

A number of interventions toward reduction of psychotropic medicines in this setting are described in the literature: from sensitization of caregivers on risks of ADRs in order to reduce new treatments (e.g., falls and tolerance development), to the introduction of non-pharmacological approaches for containing behavioral disorders in cognitive impairment syndromes (Ivanova et al., 2018; Wauters et al., 2019). However, drug therapy seems to represent the easiest and cheapest approach, especially when many old and frail patients were assisted in the same place.

Among community-dwelling patients, the most common interaction is represented by ACE-inhibitors and NSAIDs (about 12%, which could even be underestimated because of the frequent out-of-pocket use of NSAIDs). Also interactions of NSAIDs with diuretics and SSRIs counted for more than 1%. As a general recommendation, NSAID use by the Elderly should be carefully monitored because of possible antagonism with the antihypertensive effect and increased risk of bleeding. On the other hand, analgesic and antinflammatory treatment should not be precluded to this population, because of detrimental consequences in terms of general wellbeing and especially the risk of a sedentary lifestyle. As a matter of fact, nursing homes did not result in high frequency of NSAID prescriptions and relevant interactions; paracetamol, short opioid cycles and physical therapy are in fact frequently used instead.

As for bleeding risk, three additional pairs of drugs resulted as frequent: SSRIS—low dose ASA in both settings, vitamin K antagonists—PPIS, and vitamin K antagonists—SSRIS only in nursing homes. While appropriateness of antithrombotic therapy is not questionable, selection of clinically important indications for SSRIs and PPIs is warranted.

The potential interaction between antidiabetics and β-blockers is among the most frequent: it ranks third in both settings. Although this combination should be theoretically avoided due to the possible masking effect of hypoglycaemic crisis by β-blockers, its high frequency is not *per se* inappropriate and it is supported by most authoritative guidelines recommending β-blockers in diabetic patients in the case of concomitant stable heart failure or recent acute myocardial infarction. Moreover, its clinical impact is minor in well-trained diabetic patients and, even more, for patients in nursing home which are strictly monitored in levels of glycemia by nurses and doctors. This is an example of DDI which probably does not requires specific improvement initiatives, provided that high level of patient training and monitoring is maintained.

Risk of hypotensive episodes, and relevant falls and trauma, could result from the combination of calcium channel blockers with α-blockers, especially when the latter class is used against prostatic hypertrophy ignoring its strong hypotensive effect. Our findings in fact identified this combination as frequently used in both settings. When patients under antihypertensive treatment need antiprostatic medicines, strict monitoring of blood pressure values and revision of the overall drug therapy should be performed.

The last area of potential improvement in appropriate use is represented by antibacterials. Antidiabetics—fluoroquinolones and calcium channel blockers—macrolides showed 1% of prevalence in outpatients. In both situations, wide availability of potential alternative should stimulate prescribers in findings safer classes for patients already in chronic treatment for diabetes or cardiovascular disease. Implementation of software alerting on potential interactions just during prescription can be useful in this specific case.

This is a population-based study, which allows input to be provided to health policy makers rather than to help in the management of single patient. For this last aim, each single DDI should be assessed in the light of additional comorbidities (including organ failure and specific pharmacokinetic impairments) and by simultaneously considering concomitant DDIs and polypharmacy.

The main strength of this study is represented by the systematic approach in applying most valuable indicators of polypharmacy and DDIs in the Elderly, also by covering the two most representative settings of care (community-dwelling and nursing-home residents). Useful inputs for policy makers were obtained by this strategy.

Nursing home analysis allowed to cover all medications, no matter reimbursement rules, but lack of electronic recording data systems limits the number of subjects included in the analysis, because of very time-consuming data collection.

As for community-dwelling setting, a large (quite complete) population is covered, but typical limitations of drug utilization studies on prescription claims are present (missing out-of-pocket drug supplies, possible discrepancies between prescriptions and actual exposure, etc.). Lack of information on herbals and dietary supplements represents an additional limitation, since these products may cause interactions with prescribed drugs in more that 1 out of 10 elderly patients (Agbabiaka et al., 2018).

Our findings should undoubtedly be ascribed to the local setting. Nevertheless, the Emilia Romagna Region represents an Italian area with high-quality healthcare data archives and they are largely used for research and surveillance purposes (Gini et al., 2014; Trifirò et al., 2019; Corrao et al., 2020). Therefore, they could represent a reference for international comparisons and an input toward an Italian mapping of polypharmacy and DDIs especially in nursing homes.

## Conclusion

Routine monitoring of polypharmacy and possible DDIs in the older population is mandatory for local health authorities, in order to identify priorities for health policy in increasing appropriateness of drug therapy. Both quality indicators and strategies to address main concerns resulting from indicator applications are available in the literature and should be adapted to local data availability and healthcare organizations. Monitoring different settings of care in which older subjects are assisted provides very useful input for policy makers toward improvement and maintenance of quality of drug use in this population. Our findings highlight that reducing the use of benzodiazepines, NSAIDs and SSRIs, and consequent DDIs should be prioritized. Interventions should focus on alternative options for pain relief in outpatients and non-pharmacological management of insomnia and anxiety in nursing homes.

## Data Availability

The datasets presented in this article are not readily available because only aggregated data have been shared among Authors. Raw data are stored by the single participant Local Health Trust. Requests to access the datasets should be directed to elisabetta.poluzzi@unibo.it.
