# Supplementary material for: Comparing the Prevalence of Polypharmacy and Potential Drug-Drug Interactions in Nursing Homes and in the Community Dwelling Elderly of Emilia Romagna Region
Source: Front Pharmacol. 2021 Feb 11;11:624888. doi: 10.3389/fphar.2020.624888 (PMC7898059; doi:10.3389/fphar.2020.624888)
Supplement: Supplementary file 1 [file datasheet1.doc]

***Electronic Supplementary Material 2***

**Article Title:** Comparing the prevalence of polypharmacy and potential drug-drug interactions in nursing homes and in the community dwelling Elderly of Emilia Romagna Region

**Authors:** Burato S., Leonardi L., Antonazzo I.C., Raschi E., Ajolfi C., Baraghini M., Chiarello A., Delmonte V., Di Castri L., Donati M., Fadda A., Fedele D., Ferretti A., Gabrielli L., Gobbi S., Lughi S., Mazzari M., Pieraccini F., Renzetti A., Russi E., Scanelli C., Zanetti B., Poluzzi E.

**Corresponding Author:** Elisabetta Poluzzi, Department of Medical and Surgical Sciences, University of Bologna, Bologna, Italy (elisabetta.poluzzi@unibo.it)

**Table S4.** Prevalence rate for all studied DDIs, listed in decreasing order of prevalence among outpatients in 2018

| **Drug-Drug Interaction** | **Observed prevalence** |
| --- | --- |
| ACEI/sartans-NSAIDs | 7.2% |
| Alpha blockers/alpha-adrenoreceptor agonists- calcium channel blockers | 2.4% |
| antidiabetics-beta blockers | 2.3% |
| SSRIs-ASA | 2.2% |
| diuretics-NSAIDs | 1.3% |
| SSRIs- NSAIDs | 1.1% |
| antidiabetics-fluoroquinolones | 1.0% |
| Calcium channel blockers-Macrolides | 1.0% |
| Metformin-NSAIDs | 0.8% |
| Atorvastatin-Macrolides | 0.7% |
| Alpha blockers-beta blockers | 0.6% |
| vit. K antagonists-statins | 0.5% |
| Fluoroquinolones-Corticosteroids | 0.5% |
| Allopurinol-ACEIs | 0.4% |
| ACEIs/Sartans-Cotrimoxazole | 0.4% |
| vit. K antagonists-PPIs | 0.4% |
| Simvastatin-Macrolides | 0.3% |
| Allopurinol-ACEIs combinations | 0.3% |
| Allopurinol- thiazides | 0.2% |
| Atorvastatin-Amiodarone | 0.2% |
| Antidepressants-Tramadol | 0.2% |
| Vit.K antagonists-Fluoroquinolones | 0.2% |
| Beta blocking agents and thiazides-NSAIDs | 0.2% |
| Vit.K antagonists -Cephalosporins | 0.1% |
| Vit.K antagonists -SSRIs | 0.1% |
| Vit.K antagonists -Tapentadole | 0.1% |
| Vit.K antagonists -NSAIDs/ASA high dose | 0.1% |
| Atorvastatin -Diltiazem | 0.1% |
| Vit.K antagonists -Macrolides | 0.1% |
| Clopidogrel-Esomeprazole | 0.1% |
| Amiodarone-Fluoroquinolones | 0.1% |
| Beta blocking agents and other diuretics-NSAIDs | 0.1% |
| Ezetemibe+Simvastatin-Macrolides | 0.1% |
| Atorvastatin -triazole derivates | 0.1% |
| Simvastatin-Amiodarone | 0.1% |
| Clopidogrel-Omeprazole | 0.1% |
| Simvastatin-Diltiazem | 0.1% |
| Amiodarone-Macrolides | 0.1% |
| Statins-Fibrates | 0.1% |
| NSAIDs- fluoroquinolones | 0.0% |
| Antidepressants-Selegiline | 0.0% |
| Antidepressants-Fentanyl | 0.0% |
| Simvastatin - triazole derivates | 0.0% |
| Vit.K antagonists -Ezetimibe+Simvastatin | 0.0% |
| Dabigatran-Simvastatin | 0.0% |
| Allopurinol- K+ sparing diuretics | 0.0% |
| Dabigatran -Amiodarone | 0.0% |
| Sulfonylureas -Cotrimoxazole | 0.0% |
| Beta blocking agents, non selective-beta-adrenoreceptor agonists | 0.0% |
| Allopurinol- Beta blocking agents and thiazides | 0.0% |
| Rivaroxaban-Clarithromycin | 0.0% |
| Ezetimibe+Simvastatin-Amiodarone | 0.0% |
| Apixaban-Diltiazem | 0.0% |
| Digoxin-ARBs and diuretics | 0.0% |
| Lovastatin-Macrolides | 0.0% |
| SSRIs -Triptans | 0.0% |
| K+ sparing diuretics- Cotrimoxazole | 0.0% |
| Digoxin-ACEIs and diuretics | 0.0% |
| Ezetimibe+Simvastatin-Diltiazem | 0.0% |
| Digoxin-Macrolides | 0.0% |
| Vit.K antagonists -Venlafaxine | 0.0% |
| Allopurinol-low ceiling diuretics, thiazides | 0.0% |
| Vit.K antagonists-Cotrimoxazole | 0.0% |
| Verapamil-Beta blockers | 0.0% |
| Methotrexate-PPIs | 0.0% |
| Tamoxifen-SSRIs | 0.0% |
| Ezetimibe+Simvastatin- triazole derivates | 0.0% |
| Antidepressants-Rasagiline | 0.0% |
| Vit.K antagonists – triazole derivates | 0.0% |
| Tricyclic antidepressants-SSRIs | 0.0% |
| Carbamazepine-Macrolides | 0.0% |
| Litium-ACEIs | 0.0% |
| Rivaroxaban-Venlafaxine | 0.0% |
| Other antidepressants-Triptans | 0.0% |
| Litium-NSAIDs | 0.0% |
| Vit.K antagonists – Other analgesics and antipyretics | 0.0% |
| Vit.K antagonists -Fibrates | 0.0% |
| Ezetimibe+Simvastatin- Fibrates | 0.0% |
| Digoxin- K+ sparing diuretics | 0.0% |
| Phenytoin - Sertralin | 0.0% |
| Dabigatran-Venlafaxine | 0.0% |
| Dabigatran-Verapamil | 0.0% |
| Rivaroxaban-Fluconazole | 0.0% |
| Carbamazepine-Ibuprofen | 0.0% |
| Tamoxifen-Paroxetine | 0.0% |
| Tramadol-Carbamazepine | 0.0% |
| Lovastatin-Diltiazem | 0.0% |
| Litium-ARBs and diuretics | 0.0% |
| Vit.K antagonists -Valproate | 0.0% |
| Lovastatin- triazole derivates | 0.0% |
| Rivaroxaban-Phenobarbital | 0.0% |
| Digoxin-Thiazides | 0.0% |
| Dabigatran-Lovastatin | 0.0% |
| Rivaroxaban- Carbamazepine | 0.0% |
| Litium-ACEIs and diuretics | 0.0% |
| Rivaroxaban-Itraconazole | 0.0% |
| Phenytoin -Omeprazole | 0.0% |
| Apixaban- Phenobarbital | 0.0% |
| Phenytoin -Esomeprazole | 0.0% |
| Rivaroxaban-Dronedarone | 0.0% |
| Antidepressants-linezolid | 0.0% |
| Class Ia antiarrhythmics-SSRIs/Venlafaxine/Tricyclics | 0.0% |
| Phenytoin -Cotrimoxazole | 0.0% |
| Quetiapine-Warfarin | 0.0% |
| Apixaban-Rifampicin | 0.0% |
| Carbamazepine- triazole derivates | 0.0% |
| Lovastatin-Amiodarone | 0.0% |
| Dabigatran -Itraconazole | 0.0% |
| Dabigatran -Phenobarbital | 0.0% |
| Rivaroxaban-Rifampicin | 0.0% |
| Rivaroxaban-Phenytoin | 0.0% |
| Carbamazepine -Diltiazem | 0.0% |
| Digoxin- Beta blocking agents and thiazides | 0.0% |
| Dabigatran - Rifampicin | 0.0% |
| Dabigatran - Carbamazepine | 0.0% |
| Apixaban- Phenytoin | 0.0% |
| Phenytoin- Tricyclics | 0.0% |
| Azatioprine-Febuxostat | 0.0% |
| Dabigatran-Dronedarone | 0.0% |
| Apixaban-Itraconazole | 0.0% |
| Apixaban- Carbamazepine | 0.0% |
| Litium- Beta blocking agents and thiazides | 0.0% |
| Metotrexate-Cotrimoxazole | 0.0% |
| Litium- low ceiling diuretics, thiazides | 0.0% |
| Litium- K+ sparing diuretics | 0.0% |
| Carbamazepine-Verapamil | 0.0% |
| Antidepressants-Petidine | 0.0% |
| Ciclosporin -Rifampicin | 0.0% |
| Teofilline-Fluvoxamine | 0.0% |
| Dabigatran - Phenytoin | 0.0% |
| Rivaroxaban- Erythromycin | 0.0% |
| Rivaroxaban - Tacrolimus | 0.0% |
| Oxcarbamazepine-Diltiazem | 0.0% |
| Rivaroxaban-Ciclosporin | 0.0% |
| Disopiramide-Macrolides | 0.0% |
| Beta blocking agents and thiazides-NSAIDs | 0.0% |
| Simvastatin- imidazole derivates | 0.0% |
| Lovastatin- imidazole derivates | 0.0% |
| Atorvastatin- imidazole derivates | 0.0% |
| Ezetimibe+Simvastatin- imidazole derivates | 0.0% |
| Vit.K antagonists - imidazole derivates | 0.0% |
| Vit.K antagonists - Metronidazole | 0.0% |
| Oxcarbamazepine-Verapamil | 0.0% |
| Carbamazepine- imidazole derivates | 0.0% |
| Phenytoin- Fluoxetin | 0.0% |
| Phenytoin-Fluvoxamine | 0.0% |
| Antidepressants-MAO inhibitors | 0.0% |
| Ciclosporin - Rifabutin | 0.0% |
| Tacrolimus-Rifampicin | 0.0% |
| Tacrolimus- Rifabutin | 0.0% |
| Azatioprine-Allopurinol | 0.0% |
| Dabigatran-Ciclosporin | 0.0% |
| Dabigatran-Tacrolimus | 0.0% |
| Dabigatran-HIV protease inhibitors | 0.0% |
| Rivaroxaban-Ketoconazole | 0.0% |
| Rivaroxaban- HIV protease inhibitors | 0.0% |
| Ticagrelor-Clozapine | 0.0% |
| Apixaban-HIV protease inhibitors | 0.0% |
| Beta blocking agents, non selective- Adrenergics for systemic use | 0.0% |

ACEIs: Angiotensin Converting Enzyme Inhibitors; NSAIDs: Nonsteroidal Anti-Inflammatory Drugs; SSRIs: Selective Serotonin Reuptake Inhibitors; ASA: Acetyl Salicylic Acid; vit: vitamin; PPIs: Proton Pump Inhibitors; ARBs: Angiotensin II receptor blockers

**Table S5.** Prevalence rate for all studied DDIs, listed in decreasing order of prevalence among inpatients in nursing homes in 2018

| **Drug-Drug Interaction** | **Observed prevalence** |
| --- | --- |
| Anxiolytics, benzodiazepines-antidepressants | 11.9% |
| SSRI-ASA | 7.4% |
| antidiabetics-beta blockers | 5.3% |
| vit. K antagonists-PPIs | 4.1% |
| Anxiolytics, benzodiazepines-Opioids | 3.4% |
| Anxiolytics, benzodiazepines-Hypnotics | 2.8% |
| Allopurinol-ACEIs | 2.7% |
| Antidepressants-Fentanyl | 2.5% |
| Vit.K antagonists -SSRIs | 2.1% |
| Alpha blockers/alpha-adrenoreceptor agonists- calcium channel blockers | 1.6% |
| Anxiolytics, benzodiazepines-Antihistamines | 1.4% |
| vit. K antagonists-statins | 0.9% |
| Antidepressants-Tramadol | 0.9% |
| Quetiapine-Warfarin | 0.7% |
| Alpha blockers-beta blockers | 0.7% |
| Alprazolam-SSRIs | 0.6% |
| Vit.K antagonists -Valproate | 0.4% |
| Vit.K antagonists -ASA high dose | 0.4% |
| diuretics-NSAIDs | 0.3% |
| ACEI/sartans-NSAIDs | 0.2% |
| Diazepam-SSRIs | 0.2% |
| SSRI- NSAIDs | 0.2% |
| Allopurinol-ARBs and diuretics | 0.2% |
| Atorvastatin-Diltiazem | 0.2% |
| Vit.K antagonists -Cephalosporins | 0.2% |
| antidiabetics-fluoroquinolones | 0.1% |
| Vit.K antagonists -Venlafaxine | 0.1% |
| Clopidogrel-Esomeprazole | 0.1% |
| Antidepressants-Tapentadole | 0.1% |
| Vit.K antagonists -NSAIDs | 0.1% |
| Lorazepam-NSAIDs | 0.1% |
| Digoxin-ACEIs | 0.1% |
| Simvastatin-Amiodarone | 0.1% |
| Simvastatin-Diltiazem | 0.1% |
| Litium-ACEIs | 0.1% |
| Phenytoin - Sertralin | 0.1% |
| Apixaban-Diltiazem | 0.1% |
| Diazepam-Carbamazepine | 0.1% |
| ACEIs/Sartans-Cotrimoxazole | 0.1% |
| Vit.K antagonists -Macrolides | 0.1% |
| Litium- low ceiling diuretics and K+ sparing diuretics | 0.1% |
| Antidepressants-Linezolid | 0.1% |
| Fluoroquinolones-Corticosteroids | 0.1% |
| Dabigatran-Simvastatin | 0.1% |
| Allopurinol- low ceiling diuretics and K+ sparing diuretics | 0.1% |
| Tramadol-Carbamazepine | 0.1% |
| Dopaminergic antiparkinsonism agents-Metoclopramide | 0.1% |
| Alprazolam- Carbamazepine | 0.1% |
| Oxazepam-Carbamazepine | 0.1% |
| Verapamil- beta blockers | 0.0% |
| Calcium channel blockers-Macrolides | 0.0% |
| K+ sparing diuretics-Cotrimoxazole | 0.0% |
| Statins-Fibrates | 0.0% |
| Simvastatin-Macrolides | 0.0% |
| Atorvastatin-Macrolides | 0.0% |
| Antidiabetics-Cotrimoxazole | 0.0% |
| Metformin-NSAIDs | 0.0% |
| Vit.K antagonists – azole antifungals | 0.0% |
| Vit.K antagonists-Fluoroquinolones | 0.0% |
| Litium-thiazides | 0.0% |
| Tricyclic antidepressants-SSRIs | 0.0% |
| Antidepressants-Rasagiline | 0.0% |
| Antidepressants-Selegiline | 0.0% |
| Methotrexate-PPIs | 0.0% |
| Rivaroxaban- Carbamazepine | 0.0% |
| Rivaroxaban-Phenobarbital | 0.0% |
| Apixaban- Carbamazepine | 0.0% |
| Allopurinol- thiazides | 0.0% |
| Beta blocking agents, non selective-beta-adrenoreceptor agonists | 0.0% |
| Diazepam-Verapamil | 0.0% |
| Alprazolam-Verapamil | 0.0% |
| Diazepam-Diltiazem | 0.0% |
| Alprazolam-Diltiazem | 0.0% |
| Midazolam-Diltiazem | 0.0% |
| Diazepam- Phenytoin | 0.0% |
| Alprazolam- Phenytoin | 0.0% |
| Midazolom-Carbamazepine | 0.0% |
| Lorazepam- Phenytoin | 0.0% |
| Disopiramide-Macrolides | 0.0% |
| Class Ia antiarrhythmics-SSRIs/Venlafaxine/Tricyclics | 0.0% |
| Amiodarone-Fluoroquinolones | 0.0% |
| Amiodarone-Macrolides | 0.0% |
| Digoxin-Macrolides | 0.0% |
| Digoxin-Thiazides | 0.0% |
| Digoxin- K+ sparing diuretics | 0.0% |
| Digoxin- Beta blocking agents and thiazides | 0.0% |
| Digoxin-ARBs and diuretics | 0.0% |
| Beta blocking agents and other diuretics-NSAIDs | 0.0% |
| Beta blocking agents and thiazides-NSAIDs | 0.0% |
| Beta blocking agents, thiazides and other diuretics-NSAIDs | 0.0% |
| Simvastatin -imidazole derivates | 0.0% |
| Simvastatin-triazole derivates |  |
| Lovastatin- imidazole derivates | 0.0% |
| Lovastatin- triazole derivates |  |
| Lovastatin-Macrolides | 0.0% |
| Lovastatin-Amiodarone | 0.0% |
| Lovastatin-Diltiazem | 0.0% |
| Atorvastatin -imidazole derivates | 0.0% |
| Atorvastatin- triazole derivates |  |
| Atorvastatin-Amiodarone | 0.0% |
| Ezetimibe+Simvastatin- imidazole derivates | 0.0% |
| Ezetimibe+Simvastatin- triazole derivates |  |
| Ezetemibe+Simvastatin-Macrolides | 0.1% |
| Ezetimibe+Simvastatin-Amiodarone | 0.0% |
| Ezetimibe+Simvastatin- Fibrates | 0.0% |
| Ezetimibe+Simvastatin-Diltiazem | 0.0% |
| Vit.K antagonists-Cotrimoxazole | 0.0% |
| Vit.K antagonists -Fibrates | 0.0% |
| Vit.K antagonists - azole antifungals | 0.0% |
| Vit.K antagonists - Metronidazole | 0.0% |
| Litium-NSAIDs | 0.0% |
| Litium-ARBs and diuretics | 0.0% |
| Litium-ACEIs and diuretics | 0.0% |
| Litium- Beta blocking agents and thiazides | 0.0% |
| Carbamazepine-Macrolides | 0.0% |
| Carbamazepine -Diltiazem | 0.0% |
| Oxcarbamazepine-Verapamil | 0.0% |
| Carbamazepine- imidazole derivates | 0.0% |
| Carbamazepine- triazole derivates |  |
| Phenytoin- Fluoxetin | 0.0% |
| Phenytoin-Fluvoxamine | 0.0% |
| Phenytoin- Tricyclics | 0.0% |
| Phenytoin -Omeprazole | 0.0% |
| Phenytoin-Esomeprazole | 0.0% |
| Phenytoin -Cotrimoxazole | 0.0% |
| Antidepressants-Petidine | 0.0% |
| Antidepressants-Monoamine oxidase inhibitors | 0.0% |
| SSRIs -Triptans | 0.0% |
| Other antidepressants-Triptans | 0.0% |
| Ciclosporin -Rifampicin | 0.0% |
| Tacrolimus- Rifabutin | 0.0% |
| Metotrexate-Cotrimoxazole | 0.0% |
| Azatioprine-Allopurinol | 0.0% |
| Azatioprine-Febuxostat | 0.0% |
| Tamoxifen-SSRIs | 0.0% |
| Teofilline-Fluvoxamine | 0.0% |
| Dabigatran-Lovastatin | 0.0% |
| Dabigatran -Amiodarone | 0.0% |
| Dabigatran-Venlafaxine | 0.0% |
| Dabigatran-Verapamil | 0.0% |
| Dabigatran -Itraconazole | 0.0% |
| Dabigatran-Dronedarone | 0.0% |
| Dabigatran -Phenobarbital | 0.0% |
| Dabigatran - Rifampicin | 0.0% |
| Dabigatran - Carbamazepine | 0.0% |
| Dabigatran - Phenytoin | 0.0% |
| Dabigatran-Ciclosporin | 0.0% |
| Dabigatran-Tacrolimus | 0.0% |
| Dabigatran-HIV protease inhibitors | 0.0% |
| Rivaroxaban-Clarithromycin | 0.0% |
| Rivaroxaban-Venlafaxine | 0.0% |
| Rivaroxaban-Fluconazole | 0.0% |
| Rivaroxaban-Itraconazole | 0.0% |
| Rivaroxaban-Dronedarone | 0.0% |
| Rivaroxaban-Rifampicin | 0.0% |
| Rivaroxaban-Phenytoin | 0.0% |
| Rivaroxaban- Erythromycin | 0.0% |
| Rivaroxaban-Ketoconazole | 0.0% |
| Rivaroxaban- HIV protease inhibitors | 0.0% |
| Rivaroxaban-Ciclosporin | 0.0% |
| Rivaroxaban - Tacrolimus | 0.0% |
| Ticagrelor-Clozapine | 0.0% |
| Apixaban- Phenobarbital | 0.0% |
| Apixaban-Rifampicin | 0.0% |
| Apixaban- Phenytoin | 0.0% |
| Apixaban-Itraconazole | 0.0% |
| Apixaban-HIV protease inhibitors | 0.0% |
| Allopurinol- Beta blocking agents and thiazides | 0.0% |
| Carbamazepine-Ibuprofen | 0.0% |
| Beta blocking agents, non selective- Adrenergics for systemic use | 0.0% |
| Tamoxifen-Paroxetine | 0.0% |
| NSAIDs- fluoroquinolones | 0.0% |
| Domperidone- Fluoroquinolones | 0.0% |
| Domperidone- Macrolides | 0.0% |
| Midazolam-SSRIs | 0.0% |
| Diazepam-Macrolides | 0.0% |
| Alprazolam-Macrolides | 0.0% |
| Midazolam-Macrolides | 0.0% |
| Diazepam- azole antifungals | 0.0% |
| Alprazolam- azole antifungals | 0.0% |
| Midazolam- azole antifungals | 0.0% |
| Diazepam-Ketoconazole | 0.0% |
| Alprazolam-Ketoconazole | 0.0% |
| Midazolam-Ketoconazole | 0.0% |
| Midazolam-Verapamil | 0.0% |
| Diazepam-Rifampicin | 0.0% |
| Alprazolam- Rifampicin | 0.0% |
| Midazolam- Rifampicin | 0.0% |
| Midazolam- Phenytoin | 0.0% |
| Lorazepam- Rifampicin | 0.0% |
| Oxazepam-Valproate | 0.0% |

ACEIs: Angiotensin Converting Enzyme Inhibitors; NSAIDs: Nonsteroidal Anti-Inflammatory Drugs; SSRIs: Selective Serotonin Reuptake Inhibitors; ASA: Acetyl Salicylic Acid; vit: vitamin; PPIs: Proton Pump Inhibitors; ARBs: Angiotensin II receptor blockers
